# Chitosan Oligosaccharides Inhibit/Disaggregate Fibrils and Attenuate Amyloid β-Mediated Neurotoxicity

**DOI:** 10.3390/ijms160510526

**Published:** 2015-05-08

**Authors:** Xueling Dai, Wanqi Hou, Yaxuan Sun, Zhaolan Gao, Shigong Zhu, Zhaofeng Jiang

**Affiliations:** 1 Beijing Key Laboratory of Bioactive Substances and Functional Foods, Beijing Union University, Beijing 100191, China; E-Mails: xueling@buu.edu.cn (X.D.); sunxx@buu.edu.cn (Y.S.); zhaolan@buu.edu.cn (Z.G.); 2 College of Life Sciences, Capital Normal University, Beijing 100048, China; E-Mail: bluefire828@126.com; 3 Department of Physiology and Pathophysiology, Peking University School of Basic Medical Sciences, Beijing 100191, China; E-Mail: sgzhu@bjmu.edu.cn

**Keywords:** Alzheimer’s disease, amyloid-β peptide, chitosan oligosaccharides, aggregation, neurotoxicity

## Abstract

Alzheimer’s disease (AD) is characterized by a large number of amyloid-β (Aβ) deposits in the brain. Therefore, inhibiting Aβ aggregation or destabilizing preformed aggregates could be a promising therapeutic target for halting/slowing the progression of AD. Chitosan oligosaccharides (COS) have previously been reported to exhibit antioxidant and neuroprotective effects. Recent study shows that COS could markedly decrease oligomeric Aβ-induced neurotoxicity and oxidative stress in rat hippocampal neurons. However, the potential mechanism that COS reduce Aβ-mediated neurotoxicity remains unclear. In the present study, our findings from circular dichroism spectroscopy, transmission electron microscope and thioflavin T fluorescence assay suggested that COS act as an inhibitor of Aβ aggregation and this effect shows dose-dependency. Moreover, data from thioflavin T assay indicated that COS could significantly inhibit fibrils formation and disrupt preformed fibrils in a dose-dependent manner. Furthermore, the addition of COS attenuated Aβ1-42-induced neurotoxicity in rat cortical neurons. Taken together, our results demonstrated for the first time that COS could inhibit Aβ1-42 fibrils formation and disaggregate preformed fibrils, suggesting that COS may have anti-Aβ fibrillogenesis and fibril-destabilizing properties. These findings highlight the potential role of COS as novel therapeutic agents for the prevention and treatment of AD.

## 1. Introduction

Alzheimer’s disease (AD) is a progressive, age-related neurodegenerative disorder, characterized by the deposition of amyloid β (Aβ) forming senile plaques (SPs), intracellular neurofibrillary tangles (NFTs) and neuronal loss in the brain of AD patients [1,2]. Although there remains debate on whether Aβ accumulation is a cause or an effect of AD, Aβ overproduction and deposition appears to be the earliest identifiable event associated with the development of AD [3]. The fundamental pathogenic occurrence in AD is the misfolding and aggregation process of Aβ peptides, which triggers a cascade of events that result in the occurrence of amyloid plaques, neuronal degeneration and dementia [4]. To date, acetylcholinesterase inhibitors (*i.e.*, donepezil, rivastigmine, and galantamine) and the NMDA receptor antagonist (memantine) have been approved for treating patients with AD, though these therapies exhibit a modest benefit in improving cognitive deficits [5,6]. Currently, there is no effective therapeutic agent available to treat AD.

Given the growing prevalence and poor prognosis of AD, there is an urgent need to develop novel, effective therapeutic approaches that not only ameliorating the disease symptoms but also slowing down or inhibiting the underlying neurodegenerative process. Recently, particular attention has been paid to small chemical compounds that derived from natural products, due to the ease of accessibility and structural modification [7]. Chitosan oligosaccharides (COS), the hydrolysis products from chitosan that composed of linear polymers of β-1-4-linked d-glucosamine [8], were shown to have a wide range of biological activities including immunity regulation, anti-oxidant, anti-cancer and anti-inflammatory properties [9,10,11,12]. Our recent study suggested that COS could markedly attenuate oligomeric Aβ1-42-induced neurotoxicity via repression of oxidative stress and blocking Aβ-mediated phosphorylation of c-Jun *N*-terminal kinase [13]. However, no existing literature states whether COS have direct effects on Aβ aggregation and deposition, which is an important contributor to the occurrence of AD. In the present study, we investigated the potential effect of COS on preventing monomeric Aβ aggregation, dissembling preformed fibrils and Aβ-mediated neurotoxicity.

## 2. Results

### 2.1. Circular Dichroism Analysis of COS Influences on Aβ

**Figure 1:**
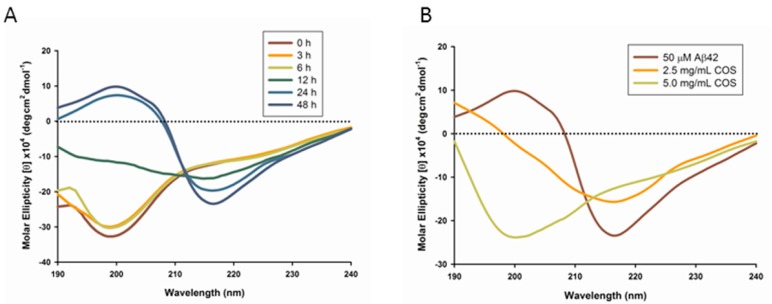
Circular Dichroism (CD) spectroscopy of Aβ42. Amyloid-β (Aβ) peptides were diluted with 10 mM phosphate buffer to a final concentration of 50 μM in each sample. (**A**) CD spectra of Aβ42 upon incubation were recorded in the UV region (190–240 nm) at 0, 3, 6, 12, 24 and 48 h respectively; (**B**) CD spectra of 50 μM Aβ42 were recorded in the UV region (190–240 nm) upon coincubation with 2.5 or 5.0 mg/mL chitosan oligosaccharides (COS) (when present) respectively for 48 h. Data were the average of six runs and represented as rainbow color curves after being smoothed.

Circular dichroism (CD) spectroscopy provides a general indicator for the increase in β-structure content that accompanies the aggregation of the Aβ peptide [14]. To investigate the structural changes of the peptide, CD spectroscopy in the far UV region (190–240 nm) was applied to detect the changes in secondary structure of Aβ1-42 (Aβ42) alone or peptides coincubated with COS for 48 h respectively. In the absence of COS, the CD spectra of Aβ exhibited a transition from unstructured random coil to β-sheet, which displayed a minimum negative band at around 216 nm upon 24 or 48 h incubation (Figure 1A). However, as shown in Figure 1B, the CD spectra of 50 μM Aβ42 coincubated with 2.5 or 5.0 mg/mL COS for 48 h suggested a largely disordered structure. The conformational conversion of 50 μM Aβ42 from physiological unfolded random coil to β-sheet structure was partly suppressed by 2.5 mg/mL COS addition, manifested by decreased negative band at ~216 nm compared to Aβ42 alone; while with addition of 5.0 mg/mL COS, the spectrum appeared a broader negative band at ~200 nm suggesting the secondary structure of Aβ is mainly random coil [15]. These results suggest that COS could prevent Aβ from converting into β-sheet structure in a dose-dependent manner, where COS at a concentration of 5.0 mg/mL was more effective than that of 2.5 mg/mL in inhibiting β-structure formation.

### 2.2. Morphologies of Aβ Aggregates Visualized by Transmission Electron Microscope

**Figure 2:**
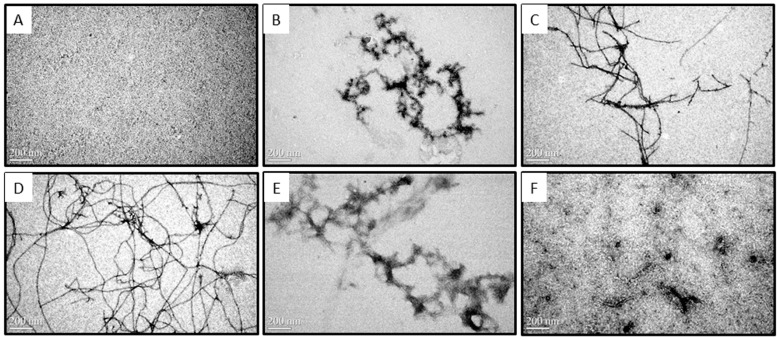
Effect of COS on Aβ aggregates visualized by Transmission Electron Microscopy (TEM). Representative TEM images of monomeric Aβ42 fractions soon after preparation (**A**) and fibrils formed by monomeric Aβ42 after 12 h (**B**), 24 h (**C**), and 48 h (**D**) of incubation at 37 °C (10 μM Aβ42, without agitation); Representative images of monomeric Aβ42 (10 μM) coincubated with 0.5 mg/mL (**E**) or 1.0 mg/mL COS (**F**) for 48 h at 37 °C were visualized by TEM. The scale bar (200 nm) is shown in the lower left of the images. Magnification: 80,000×.

Transmission electron microscope (TEM) was employed to investigate the morphologies of Aβ in the presence or absence of COS during the time course of 0–48 h at 37 °C (Figure 2). Samples from CD assay where 50 μM monomeric Aβ42 solutions in the presence or absence of 2.5 or 5.0 mg/mL COS diluted by water in the ratio of 1:5 were taken for TEM. As shown in Figure 2A, TEM analysis of monomeric fractions confirmed the absence of any aggregated Aβ in these preparations (monomeric Aβ is not detectable by TEM) [16]. After incubation at 37 °C for 12 h (Figure 2B), TEM image of 10 μM Aβ42 alone showed aggregates of various morphologies including short and branched protofibrils (10–15 nm in diameter and <200 nm in length), curvilinear aggregates and small spherical structures. Extensive long and branched fibrils with an average diameter of ~15 nm and an average length of ~1.5 μm (Figure 2C) were observed after 24 h incubation, and those aggregates prolonged into more abundant mature fibrils and produced a dense network of fibrils with an average length of ~5 μm (48 h, Figure 2D). Thus, morphology of Aβ42 alone appeared characteristic fibrillization upon incubation which is in agreement with CD results. However, the amount of fibrils appeared to decrease with increasing concentration of COS when 10 μM monomeric Aβ42 was coincubated with COS for 48 h (Figure 2E,F). 1.0 mg/mL COS (Figure 2E) was more effective than 0.5 mg/mL COS (Figure 2F) in inhibiting fibrils formation. These findings obtained from TEM images were consistent with data from CD spectroscopy, suggesting that COS may have a direct inhibitory effect on the fibrillization process of Aβ42 from its monomeric form into fibrils.

### 2.3. COS Inhibited and Disassembled Aβ Fibrils in Vitro

**Figure 3:**
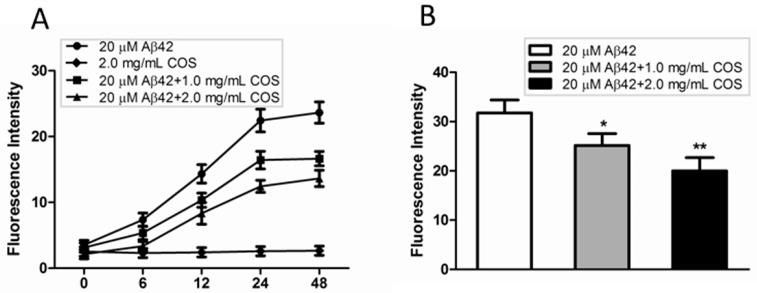
Effect of COS on Aβ fibrils formation. (**A**) Time-dependent thioflavin T (ThT) fluorescence intensity changes for 20 μM Aβ42 incubated with different concentration of COS (1.0 and 2.0 mg/mL respectively), as compared to 2.0 mg/mL COS or 20 μM Aβ42 alone; (**B**) Disaggregative effect of COS (1.0 and 2.0 mg/mL respectively) on preformed Aβ fibrils. Data were represented as mean ± SEM of three independent experiments. ** *p* < 0.01, * *p* < 0.05 *vs.* Aβ42 alone.

Thioflavin T (ThT) has been widely used as an amyloidophilic dye to detect the β-sheet structure and aggregates. To examine the inhibitory effect of COS on Aβ fibrillization, samples from CD assay where 50 μM monomeric Aβ42 solutions in the presence of 2.5 or 5.0 mg/mL COS diluted by water in the ratio of 2:5 were incubated at 37 °C for 48 h. The kinetic changes of Aβ aggregation in the presence or absence of COS were monitored. As shown in Figure 3A, the ThT fluorescence intensity of COS alone did not change during the time course of 48 h incubation, suggesting that COS had no effect on disturbing the fluorescence intensity. ThT fluorescence intensity of 20 μM Aβ42 alone increased from 0 to 48 h, and finally reached a high plateau after 48 h incubation. As expected, COS markedly reduced the ThT fluorescence intensity compared to Aβ42 alone in a dose-dependent manner, implying the inhibitory effect of COS on Aβ fibrils formation.

We also investigated whether COS could disrupt preformed Aβ fibrils using the ThT assay. As shown in Figure 3B, ThT fluorescence intensity of samples where COS were coincubated with preformed Aβ42 fibrils was significantly reduced in a concentration-dependent manner as compared with Aβ42 fibrils alone, indicating that COS could partly disaggregate the preformed Aβ42 fibrils. Taken together, these findings clearly demonstrated the effect of COS on preventing Aβ42 monomers from developing into fibrillary amyloid, and also dissembling preformed fibrils.

### 2.4. COS Attenuated Aβ42-Induced Neurotoxicity

**Figure 4:**
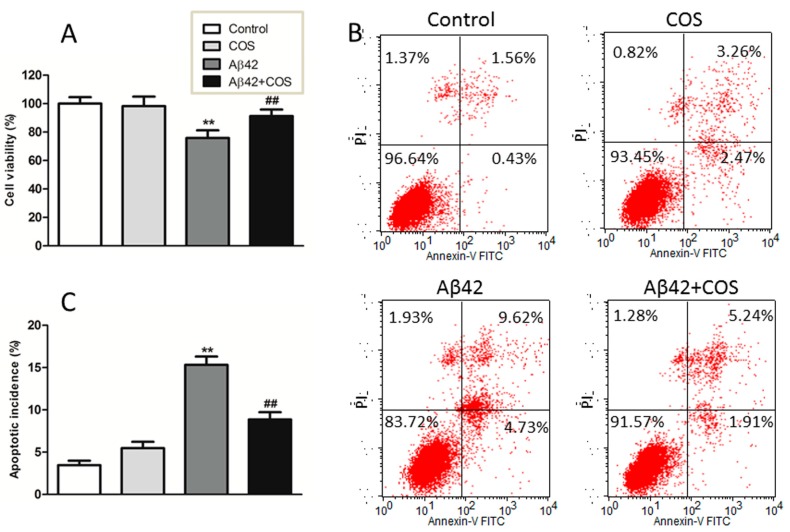
Effect of COS on Aβ42-induced neurotoxicity in cortical neurons. (**A**) Neurons were treated with 5 μM Aβ42 with or without addition of 0.5 mg/mL COS for 48 h, the cell viability was determined by the MTT assay; (**B**) Representative graphs obtained by flow cytometry using double staining with Annexin V-FITC and PI; (**C**) The apoptotic incidence of rat cortical neurons exposed to 5 μM Aβ42 in the presence or absence of 0.5 mg/mL COS for 48 h. Results were expressed as the percentage of apoptotic cells that include neurons in early and late apoptotic phases. Data were expressed as means ± SEM of three independent experiments. ** *p <* 0.01 *vs.* Control, ^##^ *p <* 0.01 *vs.* Aβ42 group.

To determine the effect of COS on Aβ42-induced toxicity, 3-[4,5-dimethylthiazol-2-yl]-2,5-diphenyltetrazolium bromide (MTT) assay was conducted to compare the cell viability of Aβ, COS, Aβ-COS on cortical neurons. As shown in Figure 4A, when neurons were incubated with COS alone at 0.5 mg/mL, no significant decline in cell viability was observed as compared to control (*p* > 0.05), suggesting that COS are nontoxic to neurons. In contrast, the cell viability of neurons exposed to 5 μM Aβ for 48 h decreased to ~75.8% of the control (*p <* 0.01), while the viability of neurons treated with both 5 μM Aβ42 and 0.5 mg/mL COS for 48 h was up to ~91.0%. These results indicated that COS markedly protected neurons from Aβ-induced neurotoxicity.

Aβ42-induced apoptosis was examined by Annexin V and PI double staining. As shown in Figure 4B, apoptotic cells were significantly increased following Aβ42 treatment for 48 h compared to that in control group, suggesting that Aβ42 treatment could lead to neuronal apoptosis. The addition of 0.5 mg/mL COS effectively rescued neurons from apoptosis induced by Aβ42 (*p <* 0.01 *vs.* 5 μM Aβ42 group), indicating that apoptosis was involving in the protective effect of COS on Aβ42-induced cytotoxicity.

## 3. Discussion

The formation of SPs in AD brain is a multiple process that involves abnormal aggregation of Aβ peptides from soluble unstructured monomers to various physical forms including β-sheet rich oligomers, protofibrils and insoluble fibrils [17]. Thus, any step in the process of Aβ production, aggregation and clearance could be considered as a potential therapeutic target [18,19]. On the other hand, since neurotoxicity is mainly associated with the formation of Aβ aggregates with β-sheet structure, the search for anti-aggregation and β-sheet disrupting compounds provides a potential therapeutic approach to treat AD [20]. Although significant efforts and progresses have been made to inhibit Aβ aggregation, reduce the existed aggregation from brain or repress the initial conformational change of Aβ into its pathological β-sheet formation, these strategies have not produced any effective pharmaceutical agent to date.

The formation of Aβ fibrils undergoes a structural transition from random coil to organized β-sheet conformation [21]. In this study, CD spectroscopy revealed that COS inhibited the aggregation of Aβ42 into β-sheet structure in a dose-dependent manner. Morphological changes of Aβ42 coincubated with COS detected by TEM were in parallel with CD spectroscopy. Further, ThT fluorescence assay confirmed that COS could effectively inhibit Aβ42 fibrils formation, and disrupt the preformed fibrils. Trehalose, a simple disaccharide that has a similar structure to chitobiose, was shown to dose-dependently inhibit Aβ40/42 aggregation and dissociate preformed aggregates partly due to the formation of H-bonds between trehalose and Aβ that suppress the interpeptide hydrogen bonding [22,23]. Properties of COS that inhibit Aβ aggregation and disassemble the preformed fibrils are likely a consequence of direct interaction with Aβ. One potential explanation for the inhibitory effect of COS can be obtained by thermodynamic reasoning. Probably, when COS were added to the monomeric peptide solutions, COS molecules altered the environment of surrounding water molecules, rendering aggregation less energetically favorable. As COS molecules would essentially isolate the water molecules surrounding the protein, thereby blocking the gain in entropy from release of this structured water layer. However, further research should be conducted to elucidate the precise mechanism by which COS interact with Aβ.

Either the oligomeric or fibrillar Aβ shows neurotoxicity stronger than its monomeric form, and mounting evidence supports toxic Aβ oligomers as the drivers of neurodegeneration [24]. Therefore, inhibiting monomeric Aβ aggregation into neurotoxic species or dissembling mature fibrils into non-toxic species is crucial to prevent and treat AD [25]. It is proposed that an efficient inhibitor should block the growth of fibrils as well as interfere with the neurotoxicity of Aβ [26]. In this study, COS coincubated with monomeric Aβ42 for 48 h significantly reduced cytotoxicity in rat cortical neurons, manifested by increased cell viability and reduced apoptotic incidence. Together, these findings indicate that the preventive effect of COS is associated with its inhibitory effects on Aβ misfolding by inhibiting Aβ aggregation formation and probably disrupting Aβ fibrils. These data extended previous work on the neuroprotective mechanism of COS against AD.

In summary, the present study along with other studies suggest the effect of COS in inhibiting aggregation and mitigating the toxicity of Aβ, which shed light on the multiple roles of COS in protecting against Aβ-associated pathology. Given the availability, low toxicity and high bio-tolerance, the use of COS as potential therapeutic agents for the treatment of AD deserves further investigation.

## 4. Materials and Methods

### 4.1. Materials

Cell culture reagents were obtained from Life Technologies (Gaithersburg, MD, USA). Amyloid β-protein fragments 1–42 (Aβ42, ≥95%), 3-[4,5-dimethylthiazol-2-yl]-2,5-diphenyltetrazolium bromide (MTT), thioflavin T (ThT), 1,1,1,3,3,3-hexafluoro-2-propanol (HFIP), dimethyl sulfoxide (DMSO) were purchased from Sigma-Aldrich (St. Louis, MO, USA). COS (average molecular weight <1000, DD. 91.3%) were obtained from Dalian Glycobio Co., Ltd. (Dalian, China). Annexin V: FITC Apoptosis Detection Kit I was obtained from BD Biosciences (San Jose, CA, USA). All other reagents were of the highest grade available and purchased from Sigma-Aldrich unless otherwise indicated.

### 4.2. Peptide Preparation

Aβ42 was obtained in lyophilized form on arrival. To disrupt the preformed Aβ aggregates into monomers, Aβ42 was initially dissolved in HFIP to a final concentration of 1 mM, sonicated for 30 min, and then centrifuged at 14,000 rpm for 20 min at 4 °C to remove any pre-existing aggregates [27]. The HFIP was allowed to evaporate in the fume hood overnight, while any remaining trace of HFIP was spin-vacuumed using a Thermo Savant Speed-Vac system (Waltham, MA, USA). The peptide film was stored at −80 °C until use.

### 4.3. Inhibition and Disruption Assay

HFIP-treated samples (homogeneous solution of Aβ monomers) were completely resuspended to 5 mM in DMSO by pipette mixing followed by bath sonication for 5 min. Aβ fibrils were prepared by diluting Aβ in DMSO to 100 μM in 10 mM HCl, vortexing for 30 s, and then centrifuged at 14,000 rpm for 30 min at 4 °C to remove any existing oligomers. The supernatant was collected for further experiments [14]. For inhibition experiments, COS stock solution was dissolved in freshly prepared Aβ42 monomer solutions to a final concentration of 2.5 and 5.0 mg/mL (with Aβ final concentration of 50 μM), or further diluted to a required concentration. For disruption assay, Aβ fibrils were prepared by incubating at 37 °C for 48 h, which is long enough to form mature fibrils. To examine the effect of COS on Aβ fibrils disruption, COS were coincubated with Aβ fibrils at 37 °C for another 48 h.

### 4.4. Circular Dichroism Spectroscopy (CD)

Secondary structural changes in peptide were detected using CD spectroscopy [28]. All measurements were performed in quartz cuvette cells with a path length of 1 mm and scanned with a J-810 CD spectropolarimeter (Jasco, Tokyo, Japan). The stock peptide was spin-vacuumed then dissolved in 10 mM phosphate buffer (pH 7.4), and the final concentration of Aβ in each sample was 50 μM. CD measurements were carried out between 190 and 240 nm using the following parameters: 2-nm bandwidth, 20 nm/min run speed, 0.5-nm step size, and 2-s response time. Direct CD measurements (θ, in mdeg) were converted to molar ellipticity, (θ) (deg·cm^2^·mol^−1^) using the formula (θ) = θ/10 × *c* × *l*, where *c* represent the molar concentration and *l* the path length (cm). Background values for each test were subtracted from the corresponding values of each sample, and the spectra were smoothed using the Jasco software FFT filter function and converted to molar ellipticity.

### 4.5. Transmission Electron Microscopy (TEM)

The morphological changes of Aβ aggregation in the presence or absence of COS were characterized by TEM. Samples used in the CD spectroscopy were diluted in 1:5 ratios and taken for TEM measurements at different time points to correlate Aβ42 morphological changes with Aβ growth kinetics. Each sample was spotted onto glow-discharged, Formvar-carbon coated 300 mesh copper grids (Ted Pella Inc., Redding, CA, USA) for 1 min, dried, and then negatively stained with 2% uranyl acetate. The excess liquid was blotted, and the grid was allowed to dry. The prepared samples were then examined with a JEM-1230 electron microscope (JEOL, Tokyo, Japan) at the voltage of 80 kV.

### 4.6. Thioflavine T (ThT) Fluorescence Assay

Aβ42 fibrillization and preformed fibrils disruption in the presence or absence of COS was monitored using ThT fluorescence assay. ThT solution was diluted in Tris-buffer (pH = 7.4) to a final concentration of 10 μM. The fluorescence intensity was measured at 37 °C using a Varioskan multimode microplate spectrophotometer (Thermo, Waltham, MA, USA) under kinetic fluorometric mode. Measurements were carried out at an excitation wavelength of 450 nm and an emission of 485 nm [28]. To account for background fluorescence, the fluorescence intensity from solution without Aβ42 was subtracted from solution containing Aβ42.

### 4.7. Primary Neuronal Culture and Treatment

This study was performed in compliance with the Public Health Service Policy on Humane Care and Use of Laboratory Animals. Cortical neuronal cultures were carried out as described previously [15]. Briefly, embryonic day 17 Sprague Dawley rat cortices were dissociated and suspended in fresh neurobasal medium plus 2% B27 supplements, then plated onto poly-d-lysine-coated 96-well culture plates at a density of 5 × 10^4^ cells per well and maintained at 37 °C in CO_2_ incubator. This method resulted in cultures highly enriched for neurons (>95% purity) as assessed by immunostaining against MAP-2. Neurons were allowed to mature for 7 days before commencing treatment. For treatment, Aβ42 samples with or without COS were diluted to a concentration of 5 µM in neurobasal medium, and finally added to the cultures for 48 h.

### 4.8. Cell Viability Assay

The cellular viability was measured by MTT reduction. Briefly, MTT solution in phosphate-buffered saline (PBS) was added to a final concentration of 0.5 mg/mL. The plate was incubated at 37 °C for additional 4 h. Finally, the medium containing MTT was removed and 100 μL DMSO was added to each well, and agitated at room temperature for 30 min to dissolve crystals. The amount of MTT formazan was determined by measuring the absorbance at 570 nm, with 630 nm as a reference.

### 4.9. Flow Cytometric Detection of Cell Apoptosis

Cell apoptosis was detected by flow cytometry using Annexin V Apoptosis Detection Kit according to the manufacturer’s protocol. Briefly, cultured neurons were washed twice by PBS, trypsinized and centrifuged at 1000 rpm for 5 min, and the pellet was resuspended in binding buffer with FITC-conjugated Annexin V and PI for 15 min at room temperature, and then analyzed by flow cytometry (FACSCalibur; BD Biosciences, San Jose, CA, USA).

### 4.10. Statistical Analysis

The results were expressed as means ± SEM of at least three independent experiments. Statistical evaluation was performed by one-way analysis of variance (ANOVA) followed by Student-Newman-Keuls as *post-hoc* test. *p* < 0.05 was considered to be statistically significant.
